# Strengthening the One Health Approach in the Eastern Mediterranean Region

**DOI:** 10.2196/41190

**Published:** 2023-03-21

**Authors:** Ekhlas Hailat, Mirwais Amiri, Nitish Debnath, Mahmudur Rahman, Md Nurul Islam, Zahida Fatima, Yousef Khader, Mohannad Al Nsour

**Affiliations:** 1 Global Health Development | Eastern Mediterranean Public Health Network Amman Jordan; 2 Development Alternatives Incorporated Dhaka Bangladesh; 3 Animal Sciences Division Pakistan Agricultural Research Council Islamabad Pakistan; 4 Department of Public Health Faculty of Medicine Jordan University of Science and Technology Irbid Jordan

**Keywords:** One Health, operationalization, zoonosis, antimicrobial resistance, Eastern Mediterranean region countries

## Abstract

One Health aims to use a multidisciplinary approach to combat health threats at animal, human, and environmental health interfaces. Among its broad focus areas are issues related to food safety, the control of zoonoses, laboratory services, neglected tropical diseases, environmental health, biosafety and biosecurity, and combatting antimicrobial resistance. A roundtable session was conducted on November 18, 2021, as part of the Eastern Mediterranean Public Health Network's (EMPHNET) seventh regional conference to highlight what role Global Health Development (GHD)|EMPHNET can play to strengthen the One Health approach. This viewpoint summarizes the findings of the roundtable discussion to highlight the experts’ viewpoints on strengthening the One Health approach, including the extent of zoonotic diseases and the dynamics of pathogens and emerging diseases; the occurrence of antimicrobial-resistant pathogens as a silent pandemic; issues surrounding the globalization of trade and food safety; the importance of integrated solutions as a new norm; issues around the institutionalization and governance toward effective operationalization of the One Health approach in the region; and how the One Health approach can be operationalized at global, regional, and local levels. The panel concluded that One Health is an integrated unifying approach that aims to sustainably balance and optimize the health of people, animals, and ecosystems, and provided recommendations to strengthen the One Health approach. It also discussed how GHD|EMPHNET can play its role in transferring the concept of One Health from theory to practice via a solid operationalization road map guide at the Eastern Mediterranean region level. The five broad priority areas of this operational guide include (1) establishing and strengthening a governance architecture, legal framework, and policy and advocacy structure for One Health operationalization in the region; (2) fostering coordination, communication, and collaboration for One Health actions across the region and beyond; (3) building the workforce capacity for effective One Health operationalization in the region; (4) supporting regional platforms for timely, effective, and efficient data sharing and exchange on all One Health–related issues; and (5) supporting risk communication, behavior change communication, and community engagement efforts in the region.

## Introduction

One Health has been defined by the World Health Organization (WHO) as “an approach to designing and implementing programs, policies, legislation, and research in which multiple sectors communicate and work together to achieve better public health outcomes” [1]. The importance of One Health is ever apparent in recent decades, as rapidly increasing human populations in the 21st century have led to encroachment into new geographic areas. Marked changes in climate and land use such as deforestation and intensive farming practices have resulted in more people living in close contact with domestic and wild animals [2]. Recent reports have revealed that more than 25% of original forest cover has been lost, and 75% of terrestrial environments and 66% of marine environments were severely altered by human interventions [3]. This close contact between animals and their environments creates increasing opportunities for the spilling over of pathogens between animals and people, and the rise of new diseases (ie, emerging infectious diseases [EIDs]). Furthermore, the movement of people, animals, and animal products has grown due to advances in international travel and trade, allowing these EIDs to spread easily across borders and around the world [4].

For effective prevention, detection, and response to EIDs or zoonotic outbreaks, communication, coordination, and collaboration among experts from all relevant fields, including public, animal, and environmental health professionals, working closely to share data and expertise is needed [1]. The WHO, Food and Agriculture Organization of the United Nations (FAO), and the World Organisation for Animal Health, formerly the Office International des Epizooties (OIE), have led the charge in promoting multisectoral responses to these issues and other public health threats at the human-animal-ecosystem interface [1,5,6].

In this regard, Global Health Development (GHD)|Eastern Mediterranean Public Health Network (EMPHNET) strongly believes in the effective role of One Health in responses and actions at the animal-human-ecosystem interface, especially targeting emerging and endemic zoonoses, and commends the role of FAO-OIE-WHO Tripartite to create and support One Health programs. A roundtable session was conducted on November 18, 2021, as part of EMPHNET’s seventh regional conference to highlight what role GHD|EMPHNET can play to strengthen the One Health approach. This viewpoint summarizes the findings of the roundtable discussion to highlight the drivers, integrated solutions, and success stories regarding the implementation of One Health, and highlight the role that GHD|EMPHNET can play in transferring the concept of One Health from theory to practice.

## Roundtable Panel Discussion

The panel members discussed the extent of zoonotic diseases and the dynamics of pathogens and emerging diseases. It was highlighted that nearly 75% of all new or EIDs affecting humans at the beginning of the 21st century are of zoonotic origin [4]. Of those, 71.8% are reported to have genetic origins from wildlife, indicating increasing spillover in recent years [7]. Examples of the effects of increasing interconnectedness and the global impact of these diseases are the HIV/AIDS, severe acute respiratory syndrome (SARS), the H5N1 strain of avian influenza, and the 2009 H1N1 influenza virus pandemics. The speed by which these diseases emerge and spread causes serious economic and developmental concerns, in addition to their effects on public health. The emergence of these diseases had been concentrated in certain “hot spot” areas, like Central Africa, South and Southeast Asia, and Latin America, where compounding factors contribute to disease spread and highlight the need for the improvement of disease detection and response capacities in these countries [8].

Several infectious viruses have emerged or re-emerged from wildlife, generating serious threats to the global health and the global economy. Ebola and Marburg hemorrhagic fevers, Lassa fever, dengue fever, yellow fever, West Nile fever, Zika, and chikungunya vector-borne diseases, swine flu, Middle East respiratory syndrome (MERS), and the recent COVID-19 are additional examples of zoonoses that have spread internationally, causing significant impact and creating a need for rapid intervention from scientists and public health professionals [9]. In fact, evidence suggests that SARS, MERS, and COVID-19 must serve as a wake-up call to be better prepared when facing the coming onslaught of the pathogen [10].

The panel also discussed the occurrence of antimicrobial-resistant pathogens as a silent pandemic. Antimicrobial resistance (AMR) occurs when bacteria, viruses, fungi, and parasites no longer respond to medications, making infections more difficult to treat [11]. AMR is a prime example of a global public health threat, which requires urgent multisectoral action [11-13]. In fact, the WHO has declared that AMR is one of the top 10 global public health threats facing humanity, citing the misuse and overuse of antibiotics as the main drivers in the development of microbial drug resistance [13]. In addition, AMR poses a significant threat to world economies, increasing mortality and disability rates, increasing longer hospital stays, and creating a need for new drug developments. The most alarming aspect of AMR is that the reduced effectiveness of antibiotics and other antimicrobials may create a future in which major surgeries and cancer chemotherapies are considered too risky [13]. While antibiotic misuse in medicine has been addressed increasingly in recent years, abuse in the agricultural sector is massively neglected and more extensive. Indeed, the Food and Drug Administration reports revealed that more than 20 million pounds of antibiotics were sold for use in livestock farms in 2014 [14] and about 80% of medically important antibiotics are regularly fed to livestock in some countries [15].

Another important topic discussed during the panel revolved around the globalization of trade and food safety. It was highlighted that globalization of trade plays an important role in disease spread and food safety, posing a challenge to the public health sector [16]. FAO promotes One Health with a focus on food safety and security, sustainable agriculture, AMR, nutrition, animal and plant health, fisheries, and livelihoods. Ensuring a One Health approach is essential for progress to anticipate (early warning), prevent, detect, and control responses to diseases that spread between animals and humans; tackle AMR; ensure food safety; prevent environment-related human and animal health threats; and combat many other health challenges arising at human-animal-environmental interfaces. Good practices from farm to fork represent a One Health approach to food safety [17].

The panel also emphasized the importance of integrated solutions as a new norm. It was highlighted that humans, animals, and environments are ever intertwined in the current globalized landscape. Therefore, a multidisciplinary approach is necessary to address any resultant emergence of zoonotic events. One Health presents a shift in the way we think about human and animal health and offers a new direction to tackle these issues, but successful implementation of interventions requires multisectoral collaboration, communication, and coordination, as well as integration. The silo mentality of certain institutions can impede the progress toward an integrated, inclusionary response and must be adjusted for effective action plans. This can be done by reframing One Health as a way to aid the smooth implementation of plans by offering a road for the cooperation of all relevant sectors and departments when handling any public health issue. When developing action plans or response programs, all concerned parties should be contacted, ideally creating a multidisciplinary team. The One Health teams can include health care providers, public health professionals, and epidemiologists representing the human health sector, and veterinarians, veterinary epidemiologists, para-veterinarians, farmers, and agriculture field workers representing animal health, in addition to ecologists and wildlife experts representing the environmental sector. One Health teams can also include law enforcement officers, social scientists, policy makers, and community members as needed to ensure effective collaboration and representation of any matter concerning the interaction at the animal-human-environment interface [18].

Another point discussed by the panel revolved around the importance of institutionalization and the governance toward effective operationalization of the One Health approach in the region. The FAO, OIE, WHO, and the United Nations Environment Program (UNEP) have advocated the new operational definition of One Health as recommended by their advisory panel, the One Health High Level Expert Panel (OHHLEP), on December 1, 2021 [19-21]. The new One Health definition developed by the OHHLEP states “One Health is an integrated, unifying approach that aims to sustainably balance and optimize the health of people, animals, and ecosystems” [18]. It is important to stress that institutionalization and governance of the One Health approach within the government systems is crucial to reflect the success of its implementation across the region. This will only be achieved if there is enough buy-in from the countries regarding the benefits of One Health application on the public, agricultural, and economic lives of their citizens and landscapes. Ultimately, the goal is to ensure that the One Health way of thinking is sustained in all governmental operations and entities working to prevent, prepare, detect, and respond to public health events and infectious diseases. This would ensure not only more efficient national action plans but also the prosperity of nations and meeting of sustainable developmental goals [21,22].

Finally, the panel discussed how the One Health approach can be operationalized at global, regional, and local levels. It was discussed that the Tripartite Zoonoses Guide (TZG), jointly developed by the FAO, OIE, and WHO to support countries in adopting a One Health approach to address zoonotic outbreaks, provides recommendations, options, and best practices, which can be used to assist countries in achieving sustainable methods for dealing with diseases with spillover potential. The TZG provides an operational guide for countries to develop the necessary capacities for preparedness for zoonotic events and efficient flow of information among concerned parties, even in low-resource settings [23]. Fortunately, One Health was adopted by the Group of Seven countries, Group of 20 countries, and World Health assemblies to reform public health, which may facilitate their implementation globally [23].

## Recommendations and Key Action or Follow-up Areas

As a result of the panel discussion and the subsequent questions raised by the participants during the questions and answers session, the most essential One Health considerations and their implications specifically pertaining to the countries across the region were identified. Recommendations to strengthen the One Health approach include institutionalization and governance of the One Health strategy, securing political commitment and influencing policy changes, developing a One Health legal framework, establishing an effective coordination mechanism and promoting multisectoral collaboration, community engagement for breaking silos, and better understanding of the interconnectedness and interdependence of human-animal-ecosystem interfaces. On the other hand, it was emphasized that epidemiological data and laboratory information should be shared across sectors to ensure effective detection of and response to health threats. Joint responses to health threats should be implemented by trained One Health workforce across sectors at the local, national, regional, and global levels.

As a success story on the active follow-up of the panel session, an important immediate next step was to collect and synthesize all important discussion points and develop an operational guide specifically geared toward the One Health priorities of the countries across the region. Thus, in April 2022, GHD|EMPHNET developed a technical guide entitled “Operationalization of the One Health Approach in the Eastern Mediterranean Region” to serve as a road map for One Health operationalization at the regional level [24]. The guide took into consideration the operational definition of One Health [19-21] and the most important themes that emerged from the panel discussion. Thus, for the effective operationalization of the One Health approach across the region, the following five broad priority areas were outlined: (1) establishing and strengthening a governance architecture, legal framework, and policy and advocacy structure for One Health operationalization in the region; (2) fostering coordination, communication, and collaboration for One Health actions across the region and beyond; (3) building the workforce capacity for effective One Health operationalization in the region; (4) supporting regional platforms for timely, effective, and efficient data sharing and exchange on all One Health–related issues; and (5) supporting risk communication, behavior change communication, and community engagement efforts in the region. Each of these five broader strategic areas contains additional subcomponents to allow for the development of country-specific implementation action plans.

As a further follow-up of the panel discussion, GHD|EMPHNET will establish a regional committee on One Health. This committee will assume two main roles or dual functions: (1) ensure effective regional communication and coordination by serving primarily as a *liaison* between the countries in the region and relevant global entities involved in the One Health efforts and (2) ensure effective regional collaboration and capacity development by functioning as a *technical advisory, support, and oversight body* across the region to facilitate the One Health Quadripartite work in the region and ensure that all stakeholders across the region are actively engaged/involved in the One Health response. Further, GHD|EMPHNET will work with the countries and relevant One Health stakeholders in the region, using its documented road map [24], to create operational work plans for the countries within and overall operational framework for the region.

## Conclusions

As promoted by the Quadripartite platform of the FAO, World Organisation for Animal Health, WHO, and UNEP, One Health is an integrated unifying approach that aims to sustainably balance and optimize the health of people, animals, and ecosystems. The health of humans, domestic and wild animals, plants, and the wider environment (including ecosystems) are closely linked and interdependent. To face new health challenges that emerge at the human-animal-environment interface, collaboration, coordination, communication, and concerted action between different sectors are needed, in addition to institutionalization and governance of the One Health approach. However, many countries lack the capacity to implement such collaboration, and international organizations, nongovernmental organizations, and private sectors can help these countries. In this context, GHD|EMPHNET can play an effective role in promoting and transferring the concept of One Health from theory to practice through its developed technical guide for the operationalization of the One Health approach at the regional level.

## Abbreviations

AMR: antimicrobial resistance
EID: emerging infectious disease
EMPHNET: Eastern Mediterranean Public Health Network
FAO: Food and Agriculture Organization of the United Nations
GHD: Global Health Development
MERS: Middle East respiratory syndrome
OHHLEP: One Health High Level Expert Panel
OIE: Office International des Epizooties
SARS: severe acute respiratory syndrome
TZG: Tripartite Zoonoses Guide
UNEP: United Nations Environment Program
WHO: World Health Organization
